# Patterns of Extrathoracic Metastasis in Lung Cancer Patients

**DOI:** 10.3390/curroncol29110691

**Published:** 2022-11-16

**Authors:** Hyung Kyu Park, Joungho Han, Ghee Young Kwon, Min-Kyung Yeo, Go Eun Bae

**Affiliations:** 1 Department of Pathology, Chungnam National University School of Medicine, Munwha-ro 266, Daejeon 35015, Republic of Korea; 2 Department of Pathology and Translational Genomics, Samsung Medical Center, Sungkyunkwan University School of Medicine, Seoul 06351, Republic of Korea

**Keywords:** lung cancer, metastasis, histological type

## Abstract

Metastasis is a major cause of death in lung cancer patients. Therefore, a deeper understanding of the metastatic mechanisms is important for developing better management strategies for lung cancer patients. This study evaluated the patterns of extrathoracic metastases in lung cancer. We retrieved data for 25,103 lung cancer patients from an institutional database and then evaluated the impacts of clinicopathologic factors on metastasis patterns. We found that 36.5% of patients had extrathoracic metastasis. Younger patients had a significantly higher extrathoracic metastasis rate in most histologic subtypes. Metastases to the bone (58.3%), central nervous system (CNS) (44.3%), liver (26.6%) and adrenal gland (18.3%) accounted for 85.5% of all extrathoracic metastases. Patients with nonmucinous adenocarcinoma had significantly higher bone metastasis rate. Patients with small cell carcinoma and large cell neuroendocrine carcinoma (LCNEC) had significantly higher liver metastasis rates. Further, patients with LCNEC also had a significantly lower bone metastasis rate, and patients with squamous cell carcinoma had a significantly lower CNS metastasis rate. Patients with multiple cancers had similar patterns of metastasis compared to patients with only lung cancer. In conclusion, different histologic subtypes of lung cancer have different metastatic patterns. Our study might help clinicians decide on follow-up strategies.

## 1. Introduction

Metastasis is the major cause of death in patients with lung cancer [1], and 40–60% of lung cancer patients already have metastases at the time of diagnosis [2,3]. Consequently, despite recent advances in lung cancer treatment, lung cancer continues to be the leading cause of cancer-related death worldwide [4]. Therefore, a deeper understanding of the metastatic mechanisms is key to developing better management strategies and achieving long-term survival of lung cancer patients.

In the field of metastasis research, an important but unsolved question is what makes tumor cells prefer specific organs to metastasize [5]. The concept of organ-specific metastasis was first suggested by Stephan Paget in 1889 [6]. He compared the relationship between the tumor cell and target organ to that between seed and soil, stating: “When a plant goes to seed, its seeds are carried in all directions; but they can only live and grow if they fall on congenial soil.” [6]. This “seed and soil” hypothesis has been challenged and refined by many researchers and is now widely accepted [7,8,9]. However, we still do not fully understand what makes tumor cells choose a specific organ to metastasize.

Lung cancer has several histologic subtypes. Each subtype has a distinct morphology, clinical presentation and gene mutation profile [10]. Therefore, identifying the metastatic pattern of each lung cancer subtype may help identify factors driving organ-specific metastasis. Several previous studies have compared the metastatic patterns of different lung cancer subtypes [11,12,13,14,15]. However, most of these studies are based on small numbers of patients or population-based cancer registries. In our opinion, a study based on well-documented medical records from a single institution would be more informative and reliable.

Therefore, we performed a large, retrospective, single-institution cohort study evaluating the impact of histologic subtypes and other clinical factors on the metastatic pattern of lung cancer.

## 2. Materials and Methods

### 2.1. Patient Selection

Data from 26,589 patients diagnosed with primary lung malignancy between 1994 and 2014 were retrieved from the Samsung Medical Center Cancer Registry. Retrieved data included age at diagnosis, sex, International Classification of Diseases for Oncology, 3rd Edition (ICD-O-3) histology codes, histological diagnosis, history of other malignancy, presence of metastasis, sites of distant metastasis, presence of recurrence, and sites of recurrence. Only cases with pathologically confirmed diagnoses were included. A total of 1470 patients with uncertain histological diagnosis or a diagnosis of carcinoid, lymphoid malignancy or sarcoma were excluded. In addition, 16 patients younger than 20 years were also excluded. Finally, 25,103 patients were included. The sites of metastasis or recurrence were reviewed and reclassified for each organ. Recurrences in distant organs were considered as metastases in this study. Lung-to-lung metastasis was excluded from analysis because it is very difficult to differentiate between lung-to-lung metastatic lesions and second primary lesions.

### 2.2. Statistical Analysis

The normality of continuous variables was evaluated using the Kolmogorov–Smirnov test. Continuous variables without normal distribution are described as the median and interquartile range and were compared using the Mann–Whitney *U*-test. Categorical variables were compared using the Pearson chi-square test or Fisher’s exact test, as appropriate.

Statistical analyses were performed using OriginPro version 2021 (OriginLab, Northampton, MA, USA) and SPSS Version 17.0 (SPSS Inc., Chicago, IL, USA).

## 3. Results

### 3.1. Patient Characteristics

A total of 25,103 cases were included in this study. We categorized these cases according to their histologic diagnoses as follows: 12,878 adenocarcinomas (ADCs), 6655 squamous cell carcinomas (SCCs), 126 adenosquamous carcinomas (ASCs), 785 salivary gland-type carcinomas (SGCs), 225 sarcomatoid carcinomas (SCs), 1713 non-small cell lung cancers, not otherwise specified (NSCLCs, NOS), 2373 small cell lung cancers (SCLCs) and 350 large cell neuroendocrine carcinomas (LCNECs). Of the 12,878 ADC cases, 12,455 were categorized as non-mucinous adenocarcinomas (N-MACs) and 421 were categorized as mucinous adenocarcinomas (MACs). The 225 SC cases included 220 pleomorphic carcinomas and 5 carcinosarcomas. Patient characteristics according to histologic diagnoses are summarized in Table 1.

Among the 25,103 cases, 9153 (36.5%) had at least one extrathoracic metastasis. NSCLC, NOS had the highest extrathoracic metastasis rate (52.1%), followed by SCLC (51.7%), LCNEC (46.6%), N-MAC (40.9%), SC (40.0%), ASC (35.7%), SCC (21.7%) and SGC (18.1%). MAC had the lowest extrathoracic metastasis rate (13.8%).

Patients with metastasis were significantly younger than those without metastasis in all histologic groups, except for the MAC, SGC and LCNEC groups (Table 2). In the MAC and LCNEC groups, patients with metastasis were younger than patients without metastasis, but it was not statistically significant (*p* = 0.174 and *p* = 0.592, respectively). In the SGC group, patients with metastasis and patients without metastasis were similar in age (*p* = 0.908). In addition, female patients showed a significantly higher rate of metastasis than male patients in the major histologic groups, including the N-MAC (*p* = 0.048), SCC (*p* < 0.001), NSCLC, NOS (*p* < 0.001), and SCLC (*p* = 0.034) groups. In the other groups, sex was not significantly associated with metastasis (Table 2).

### 3.2. Metastatic Patterns

**Figure 1:**
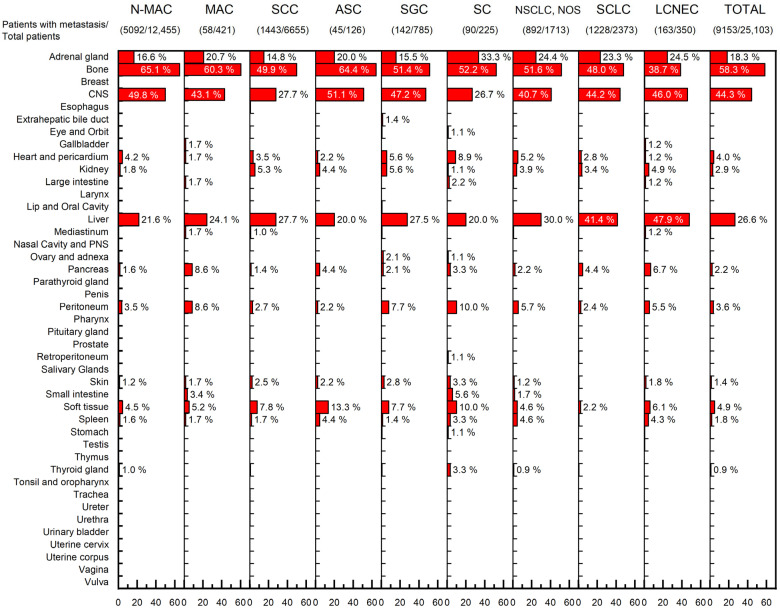
Distributions of metastases based on the lung cancer histological subtype. Each percentage indicates the percentage of patients with metastasis to the indicated organ, divided by the total number of patients with metastasis. (Abbreviations: N-MAC, non-mucinous adenocarcinoma; MAC, mucinous adenocarcinoma; SCC, squamous cell carcinoma; ASC, adenosquamous carcinoma; SGC, salivary-gland type carcinoma; SC, sarcomatoid carcinoma; NSCLC, non-small cell lung cancer; NOS, not otherwise specified; SCLC, small cell lung cancer; LCNEC, large cell neuroendocrine carcinoma; CNS, central nervous system; PNS, paranasal sinus).

A total of 15,785 metastases were found in the 9153 lung cancer patients with metastasis. The most common site of metastasis was the bone (58.3%, 5332/9153), followed by the central nervous system (CNS) (44.3%, 4057/9153), liver (26.6%, 2435/9153) and adrenal gland (18.3%, 1675/9153). Regardless of the histologic subtype, most metastases were found in these four organs; metastases to these organs accounted for 85.5% (13,499/15,785) of all metastases. Distributions of metastases according to histologic type are illustrated in Figure 1.

**Figure 2:**
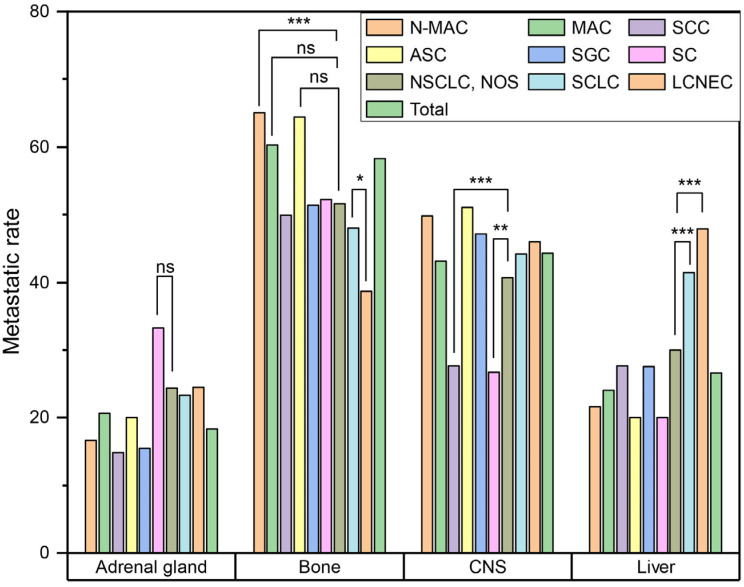
Comparison of metastatic pattern according to histological subtype. (ns, *p* > 0.05; *, *p* ≤ 0.05; **, *p* ≤ 0.01; ***, *p* ≤ 0.001) (Abbreviations: N-MAC, non-mucinous adenocarcinoma; MAC, mucinous adenocarcinoma; SCC, squamous cell carcinoma; ASC, adenosquamous carcinoma; SGC, salivary-gland type carcinoma; SC, sarcomatoid carcinoma; NSCLC, non-small cell lung cancer; NOS, not otherwise specified; SCLC, small cell lung cancer; LCNEC, large cell neuroendocrine carcinoma; CNS, central nervous system).

Next, we compared rates of metastasis to the adrenal gland, bone, liver and CNS to see whether there were significant differences in the metastatic pattern according to histologic type (Figure 2). SC had the highest rate of adrenal gland metastasis (33.3%, 30/90), but it was not statistically significant (*p* = 0.064). N-MAC had a significantly higher rate of bone metastasis than other histologic subtypes (65.1%, 3316/5092) (*p* < 0.001). MAC (60.3%, 35/58) and ASC (64.4%, 29/45) also had higher rates of bone metastasis than other histologic subtypes, but they were not statistically significant (*p* = 0.195 and 0.092, respectively). SCLC (41.4%, 508/1228) and LCNEC (47.9%, 78/163) showed significantly higher rates of liver metastasis than other histologic subtypes (both *p* < 0.001). In contrast, LCNEC (38.7%, 63/163) had a significantly lower rate of bone metastasis than other histologic subtypes (*p* < 0.001). SCC (27.7%, 400/1443) and SC (26.7%, 24/90) had significantly lower rates of CNS metastasis than other histologic subtypes (*p* < 0.001 and *p* = 0.009, respectively).

We calculated odds ratios to determine whether metastasis to a given organ could influence metastasis to another specific organ. In the entire cohort, most organs’ metastases were negatively correlated with metastasis to other organs (Supplementary Figure S1). However, metastasis to the adrenal gland was positively correlated with metastasis to the liver and soft tissue. We also performed this analysis in each histologic group. Although the small number of patients made it difficult to achieve statistical significance, the results were similar to those in the entire cohort (Supplementary Figure S1). Additionally, bone and liver metastases were positively correlated in the N-MAC and SCLC groups, and adrenal gland and bone metastases were positively correlated in the MAC group.

Last, we compared the metastatic pattern between patients with only lung cancer and patients with lung cancer and another cancer. Among 25,103 patients, 2370 (9.4%) had another carcinoma, including 373 with gastric cancer, 268 with colorectal cancer, 221 with thyroid cancer, 132 with liver cancer, 104 with prostate cancer, 90 with breast cancer, and 1183 with other carcinomas. Although the number of patients was insufficient for statistical analysis, we observed metastatic patterns in patients with multiple cancers similar to those in patients with only lung cancer (Supplementary Figure S2).

## 4. Discussion

Metastasis is a complex and dynamic process mediated by numerous factors [16]. Therefore, examining the process of metastasis is difficult. Furthermore, collecting the clinical data of metastasis is a time-consuming and error-prone process that requires significant resources. Consequently, despite its importance, metastasis is still poorly understood. Therefore, we collected and analyzed the metastatic patterns of the lung cancer in 25,103 patients based on hospital records. Previous studies have included only several hundreds to thousands of patients or were based on population-based databases [11,12,13,14,15,17,18]. To our knowledge, our study includes the largest number of patients retrieved from well-established hospital records.

The NSCLC, NOS group showed the highest metastasis rate (52.1%), which was higher even than that of the SCLC group (51.7%). This result is consistent with those of several previous studies, which suggested that the diagnosis of NSCLC, NOS is a poor prognostic factor on its own [19,20]. However, this should be interpreted with caution. NSCLC, NOS is diagnosed when the pathologist cannot further subtype NSCLC based on the histologic evaluation of the biopsied specimen [10]. If a patient with NSCLC, NOS has a resectable tumor, the diagnosis is changed after pathologic examination of the resected specimen. Consequently, only patients with unresectable, poorly differentiated NSCLC, who are more likely to have a poor prognosis, would retain a diagnosis of NSCLC, NOS throughout their treatment course. Therefore, this could cause a bias in statistical analysis. 

Among the remaining histologic groups, the SCLC group (51.7%) and the LCNEC group (46.6%) had higher metastasis rates than the N-MAC group (40.9%). The SCC group (21.7%) had a significantly lower metastasis rate than the other major histologic subtype groups. These findings are consistent with those of several previous studies [11,13]. Additionally, the SGC group (18.1%) and the MAC group (13.8%) had the second lowest and lowest metastasis rates, respectively. Because we excluded lung-to-lung metastasis in this study, these results are also consistent with those of previous studies [13,21,22].

Interestingly, younger patients had a higher metastasis rate than older patients in all histologic groups except the SGC group. Several previous studies also reported higher metastasis rates in younger patients with lung cancer [11,13,17,23]. There was no association between age and metastasis in the SGC group. This may be because the SGC group had the youngest median age and the second lowest metastasis rate.

Additionally, female patients had significantly higher metastasis rates than male patients in the major histologic groups, including in the N-MAC, SCC, NSCLC, NOS and SCLC groups. To our knowledge, no previous study has reported a higher metastasis rate in female lung cancer patients. Riihimäki et al. reported that female patients had a significantly higher rate of CNS metastasis [11]; however, it was not found to be significant in another study [13]. Therefore, the association between sex and metastasis is still inconclusive and requires further evaluation.

The bone, CNS, liver and adrenal gland were common sites of distant metastasis in all histologic groups. These four sites accounted for 85.5% (13,499/15,785) of all metastases. However, lung cancer subtypes also showed significant differences with regard to metastasis to those four organs. N-MAC had a significantly higher bone metastasis rate than other subtypes, whereas SCLC and LCNEC had significantly higher liver metastasis rates. LCNEC had a significantly lower bone metastasis rate than other subtypes, and SCC and SC had significantly lower CNS metastasis rates. A high bone metastasis rate of N-MAC [11,18], high liver metastasis rate of SCLC [11,12,13,24], and low CNS metastasis rate of SCC [11,13,23] have been previously reported. Although a previous study reported that SCLC had a higher CNS metastasis rate than other major subtypes, it was not significant in our study or another previous report [11,13]. Because previous reports only include common histologic subtypes, our findings about the metastatic patterns of the LCNEC and SC groups could not be compared to the results of previous reports.

Wang et al. reported that bone metastasis tends to co-occur with liver or distant lymph node (DL) metastasis, and liver metastasis tends to co-occur with DL metastasis [13]. We were only able to confirm a positive association between bone and liver metastases in the N-MAC and SCLC groups. They were negatively associated in the SCC group, and the association was statistically not significant in the other histologic groups. Because our study did not include DL metastasis, we could not evaluate the association between DL metastasis and other organ metastases. Additionally, we found positive associations between adrenal gland and liver metastases and adrenal gland and soft tissue metastases. Because the previous study did not include adrenal gland or soft tissue metastases, these associations were not reported.

To examine whether another carcinoma could influence the metastatic pattern of lung cancer, we compared metastatic patterns between patients with only lung cancer and patients with lung and other cancers. If metastatic organotropism is only mediated by intracellular factors, there would be no interaction between different carcinomas. However, Hoshino et al. previously reported that tumor-derived exosomes taken up by organ-specific cells prepare the pre-metastatic niche [25]. According to their hypothesis, exosomes from tumor cells select where tumor cells metastasize. They also showed that treatment with exosomes from lung-tropic models redirected the metastasis of bone-tropic tumor cells. Therefore, we hypothesized that if exosomes play a major role in determining where cancer cells metastasize, patients with multiple carcinomas could have different patterns of metastasis than patients with a single carcinoma. However, we saw no significant difference in metastatic patterns between patients with only lung cancer and those with lung and other cancers. However, it should be emphasized that our study has limitations. Although our study included 25,103 patients, only 2370 patients had a history of another carcinoma. When we divided these 2370 patients according to the other carcinoma, the largest group included only 373 patients. Further, among these 373 patients, only 41 (11.0%) had at least one metastasis of lung cancer. Consequently, the number of patients was insufficient for statistical analysis. In addition, most patients with multiple carcinomas had metachronous tumors. Because many carcinomas recur even after complete resection, it would be reasonable to assume that clinically undetectable tumor cells frequently remain in the patient’s body. Therefore, even though patients had metachronous tumors, they may have had multiple types of tumor cells that were secreting exosomes. Examining the role of exosomes in directing metastasis in patients with synchronous cancers would be more ideal.

## 5. Conclusions

In conclusion, all histologic types of lung cancer showed a strong predilection of metastasis to the bone, CNS, liver and adrenal gland. However, there were significant differences in preference among these four organs based on the histologic subtype.

## Figures and Tables

**Table 1:** Summarized clinical characteristics of the 25,103 patients included in this study.

	ADC		SCC	ASC	SGC	SC	NSCLC, NOS	SCLC	LCNEC	Total
	N-MAC	MAC								
Total, *n* (%)	12,455 (49.6)	421 (1.7)	6655 (26.5)	126 (0.5)	785 (3.1)	225 (0.9)	1713 (6.8)	2373 (9.5)	350 (1.4)	25,103 (100)
Age, median (IQR)	61 (53–69)	62 (54–70)	66 (60–72)	62 (55–68)	59 (50–67)	64 (54–70)	65 (56–71)	65 (58–70)	65 (60–71)	63 (56–70)
Sex, *n* (%)										
Male	6849 (55.0)	196 (46.6)	6162 (92.6)	92 (73.0)	399 (50.8)	177 (78.7)	1344 (78.5)	2059 (86.8)	310 (88.6)	17,594 (70.1)
Female	5606 (45.0)	225 (53.4)	493 (7.4)	34 (27.0)	386 (49.2)	48 (21.3)	369 (21.5)	314 (13.2)	40 (11.4)	7515 (29.9)
Metastasis, *n* (%)										
Absent	7363 (59.1)	363 (86.2)	5212 (78.3)	81 (64.3)	643 (81.9)	135 (60.0)	821 (47.9)	1145 (48.3)	187 (53.4)	15,950 (63.5)
Present	5092 (40.9)	58 (13.8)	1443 (21.7)	45 (35.7)	142 (18.1)	90 (40.0)	892 (52.1)	1228 (51.7)	163 (46.6)	9153 (36.5)
Number of organs with metastasis, *n* (%)										
1–3	4786 (94.0)	56 (96.6)	1384 (95.9)	40 (88.9)	127 (89.4)	81 (90.0)	824 (92.4)	1163 (94.7)	146 (89.6)	8607 (94.0)
4–6	301 (5.9)	2 (3.4)	59 (4.1)	4 (8.9)	13 (9.2)	8 (8.9)	64 (7.2)	64 (5.2)	17 (10.4)	532 (5.8)
7+	5 (0.1)	0 (0)	0 (0)	1 (2.2)	2 (1.4)	1 (1.1)	4 (0.4)	1 (0.1)	0 (0)	14 (0.2)
History of another malignancy										
No	11,279 (90.6)	347 (82.4)	5936 (89.2)	117 (92.9)	662 (84.3)	198 (88.0)	1635 (95.4)	2251 (94.9)	308 (88.0)	22,733 (90.6)
Yes	1176 (9.4)	74 (17.6)	719 (10.8)	9 (7.1)	123 (15.7)	27 (12.0)	78 (4.6)	122 (5.1)	42 (12.0)	2370 (9.4)

ADC, adenocarcinoma; N-MAC, non-mucinous adenocarcinoma; MAC, mucinous adenocarcinoma; SCC, squamous cell carcinoma; ASC, adenosquamous carcinoma; SGC, salivary-gland type carcinoma; SC, sarcomatoid carcinoma; NSCLC, non-small cell lung cancer; NOS, not otherwise specified; SCLC, small cell lung cancer; LCNEC, large cell neuroendocrine carcinoma; IQR, interquartile range; LCa, lung cancer; Dx, diagnosis.

**Table 2:** Relationship of metastasis with age and sex.

		Age in Years, Median (IQR)			Sex, *n* (%)				
		Absent	Present	*p*	Absent		Present		*p*
					Male	Female	Male	Female	
ADC	N-MAC	62 (54–69)	60 (52–67)	**<0.001**	3995 (54.3)	3368 (45.7)	2854 (56.0)	2238 (44.0)	**0.048**
	MAC	63 (55–70)	60.5 (53–67)	0.174	163 (44.9)	200 (55.1)	33 (56.9)	25 (43.1)	0.089
SCC		66 (60–72)	65 (58–70)	**<0.001**	4883 (93.7)	329 (6.3)	1279 (88.6)	164 (11.4)	**<0.001**
ASC		64 (57–70)	58 (52–66)	**0.029**	63 (77.8)	18 (22.2)	29 (64.4)	16 (35.6)	0.106
SGC		59 (50–66)	59 (51–67)	0.908	319 (49.6)	324 (50.4)	80 (56.3)	62 (43.7)	0.147
SC		65 (58–72)	61 (51–69)	**0.001**	111 (82.2)	24 (17.8)	66 (73.3)	24 (26.7)	0.111
NSCLC, NOS		66 (59–72)	63 (55–70)	**<0.001**	674 (82.1)	147 (17.9)	670 (75.1)	222 (24.9)	**<0.001**
SCLC		65 (59–71)	64 (58–70)	**0.036**	976 (85.2)	169 (14.8)	1083 (88.2)	145 (11.8)	**0.034**
LCNEC		66 (61–71)	65 (60–71)	0.592	166 (88.8)	21 (11.2)	144 (88.3)	19 (11.7)	0.900
Total		64 (57–71)	62 (54–69)	**<0.001**	11,350 (71.2)	4600 (28.8)	6238 (68.2)	2915 (31.8)	**<0.001**

Bolded text indicates statistical significance at the 0.05 level. IQR, interquartile range; ADC, adenocarcinoma; N-MAC, non-mucinous adenocarcinoma; MAC, mucinous adenocarcinoma; SCC, squamous cell carcinoma; ASC, adenosquamous carcinoma; SGC, salivary-gland type carcinoma; SC, sarcomatoid carcinoma; NSCLC, non-small cell lung cancer; NOS, not otherwise specified; SCLC, small cell lung cancer; LCNEC, large cell neuroendocrine carcinoma.

## Data Availability

The data presented in this study are available on request from the corresponding author.

## Supplementary Materials

The following supporting information can be downloaded at https://www.mdpi.com/article/10.3390/curroncol29110691/s1: Figure S1: Odds ratios of metastasis to different metastatic organs; Figure S2: Comparison of metastatic patterns between patients with only lung cancer and patients with lung cancer and history of another carcinoma. Each percentage indicates the percentage of patients with metastasis to the selected organ, divided by the total number of patients with metastasis.
