# Supplementary figures and images for: Patterns of Extrathoracic Metastasis in Lung Cancer Patients
Source: Curr Oncol. 2022 Nov 16;29(11):8794–801. doi: 10.3390/curroncol29110691 (PMC9689009; doi:10.3390/curroncol29110691)

N-MAC

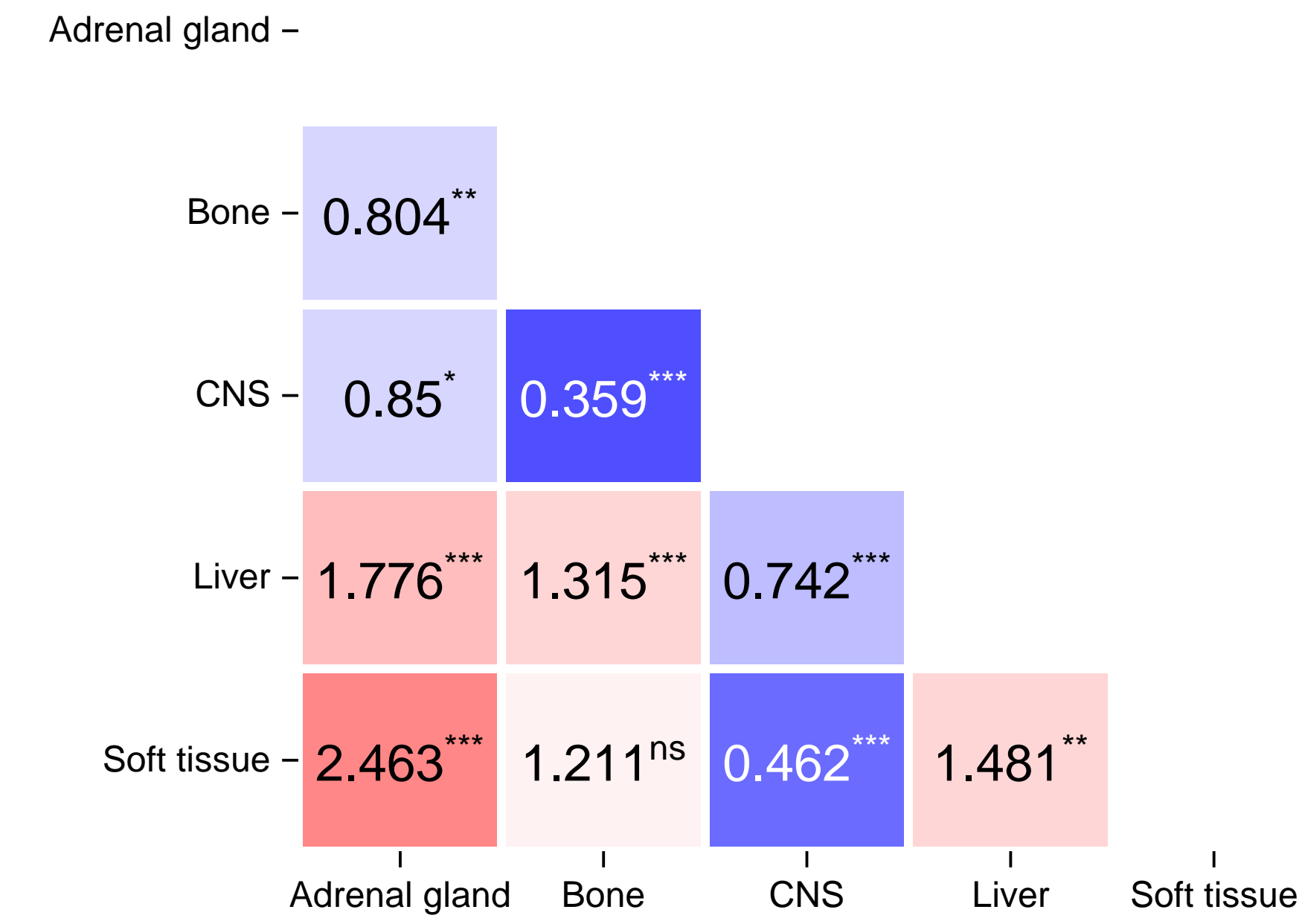

MAC

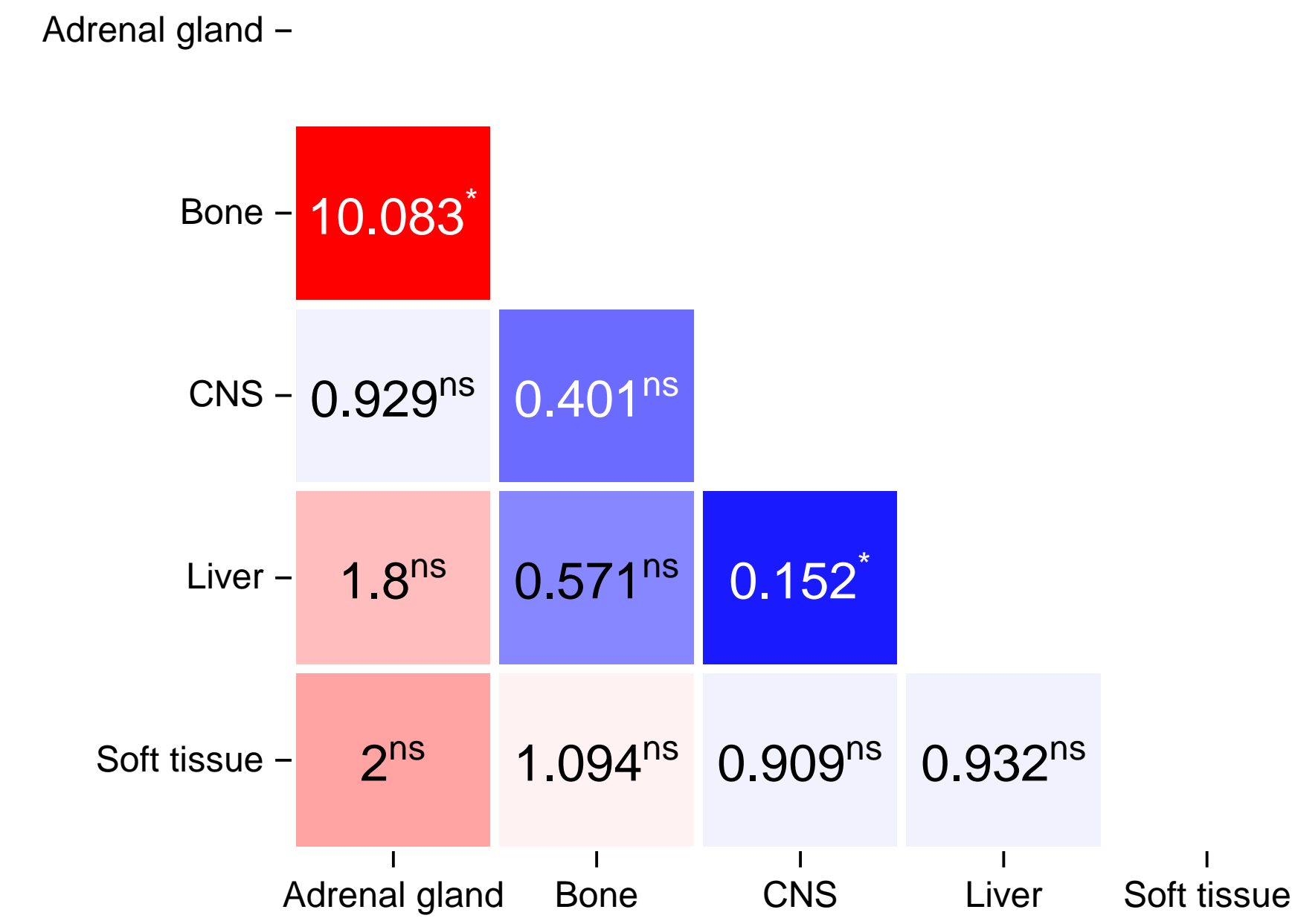

SCC

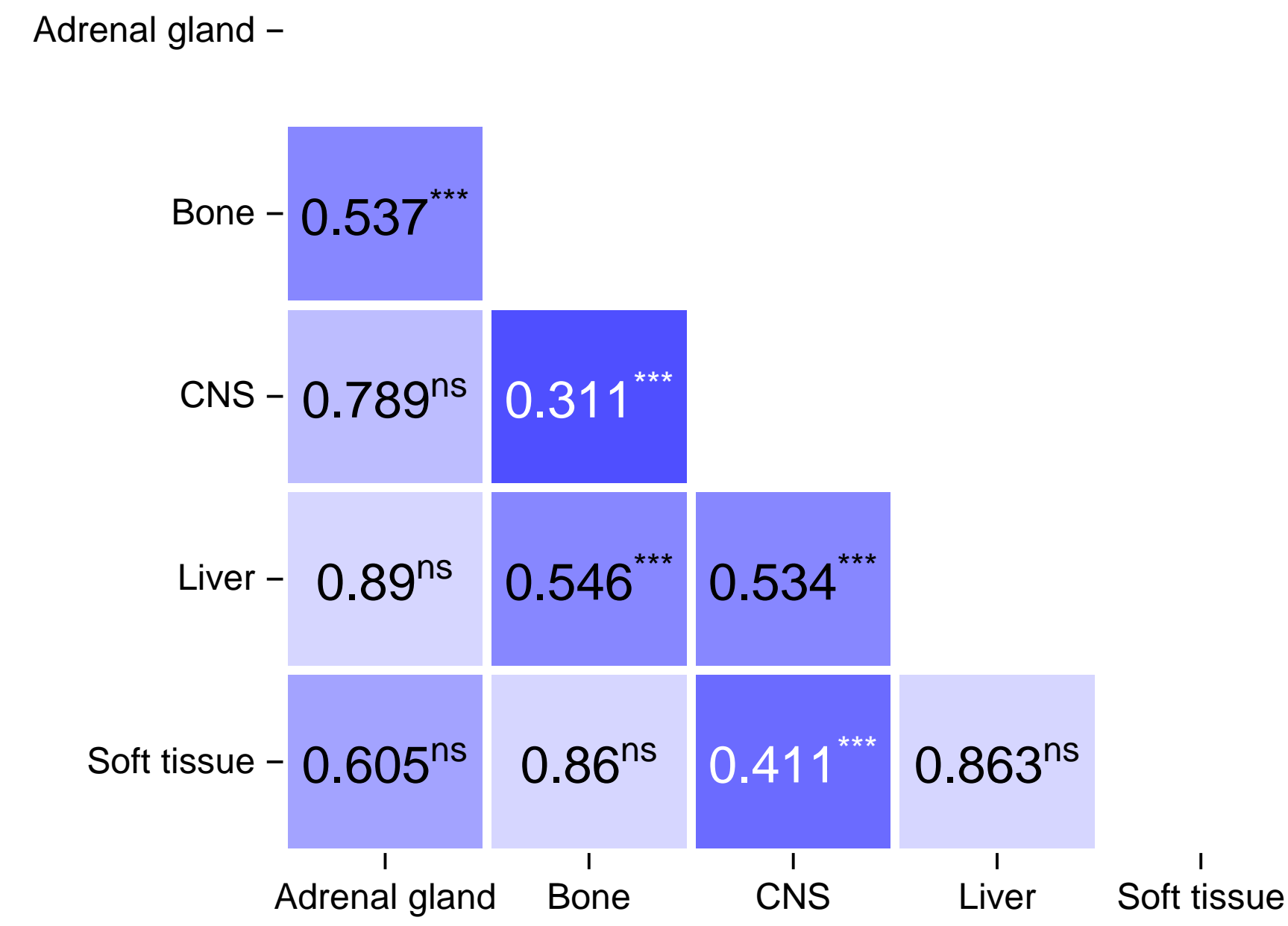

ASC

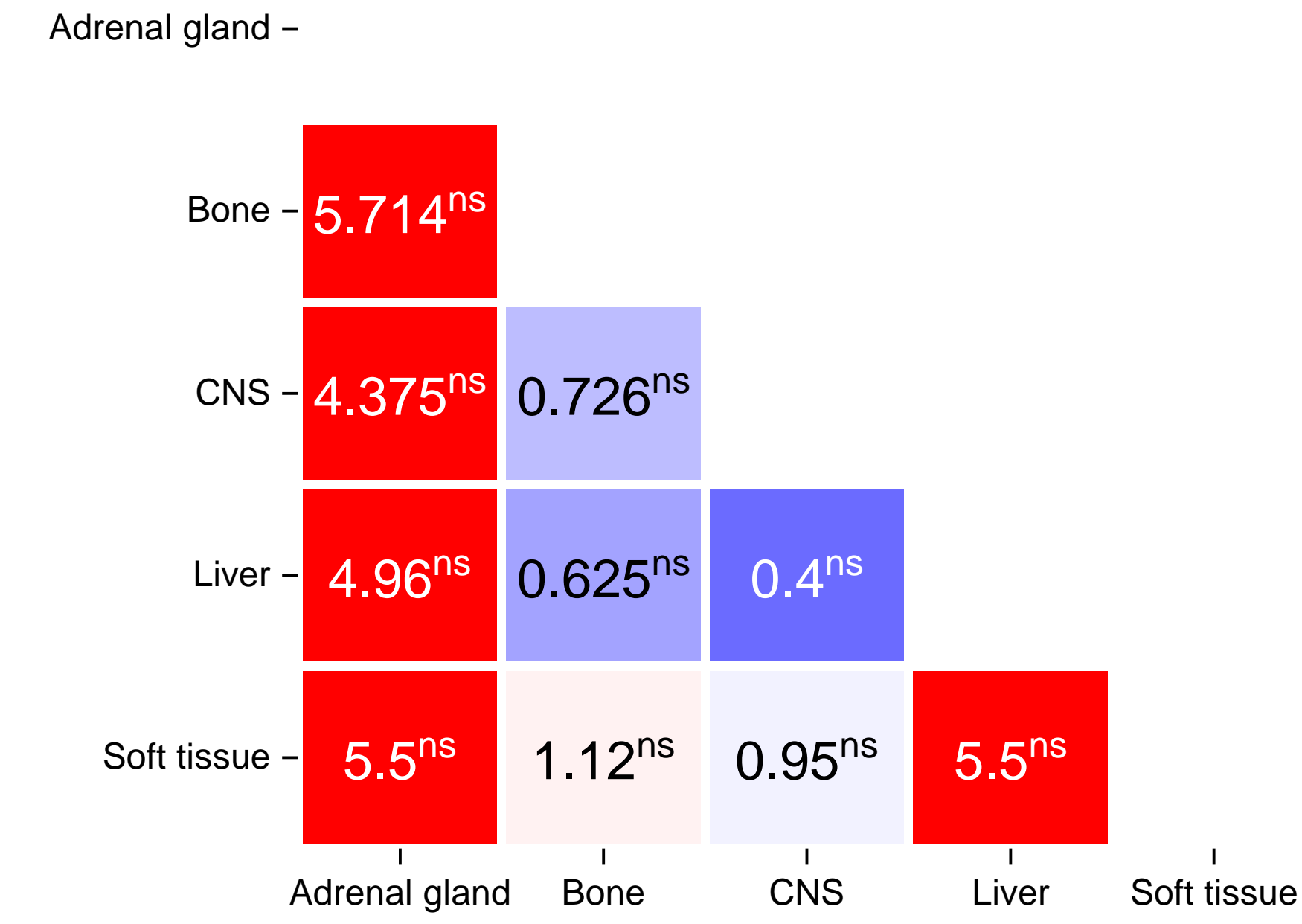

SGC

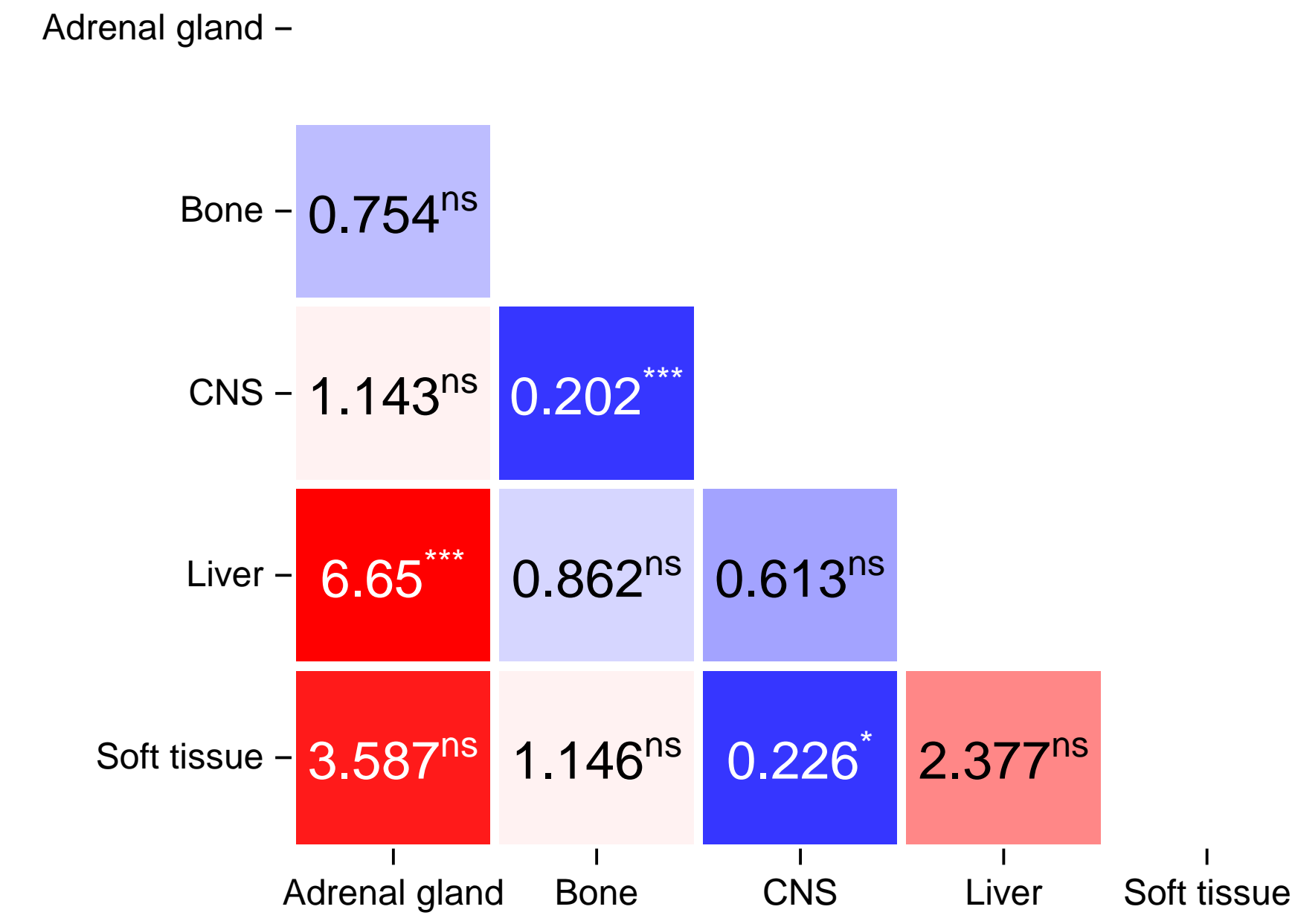

SC

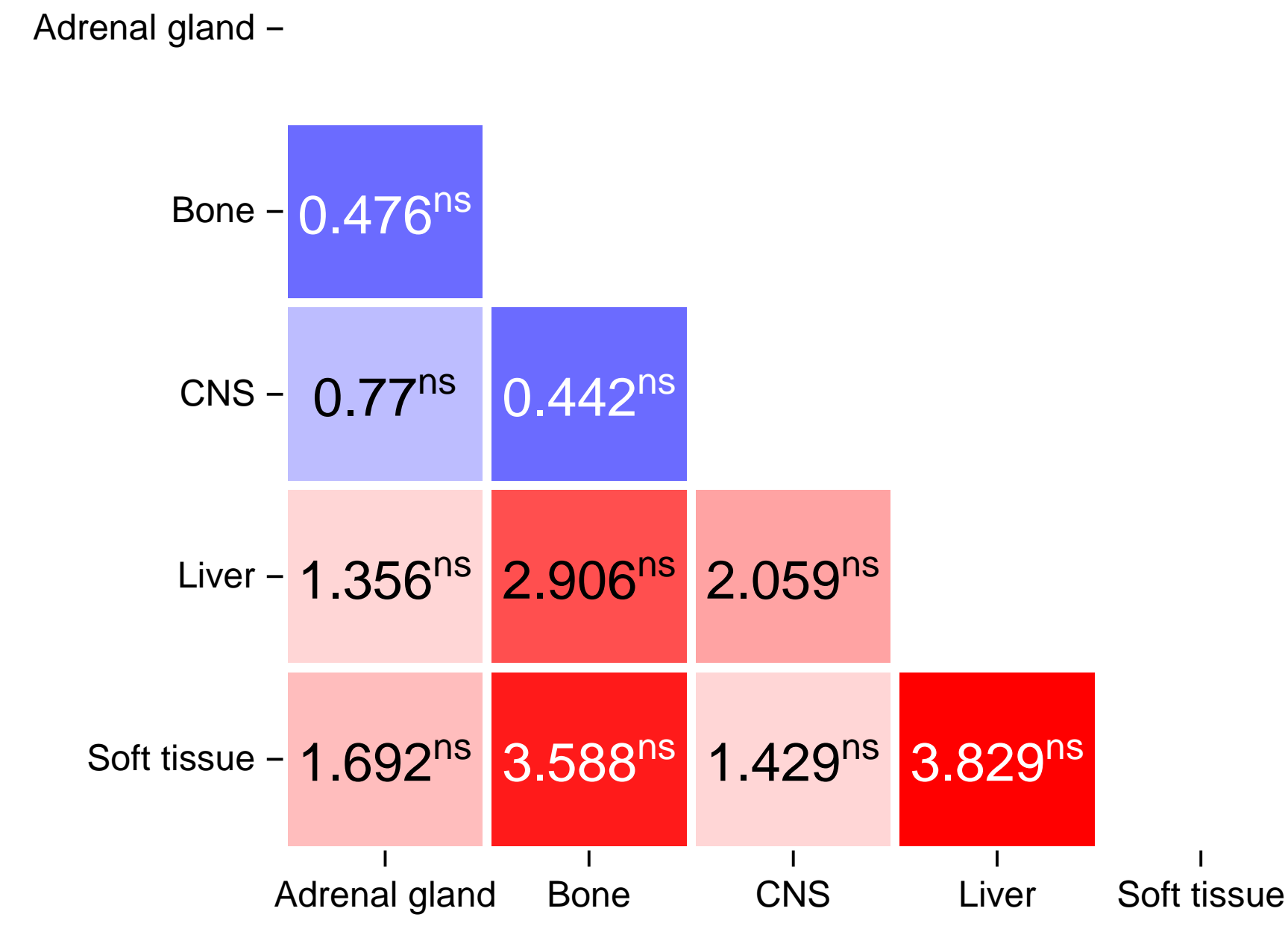

NSCLC, NOS

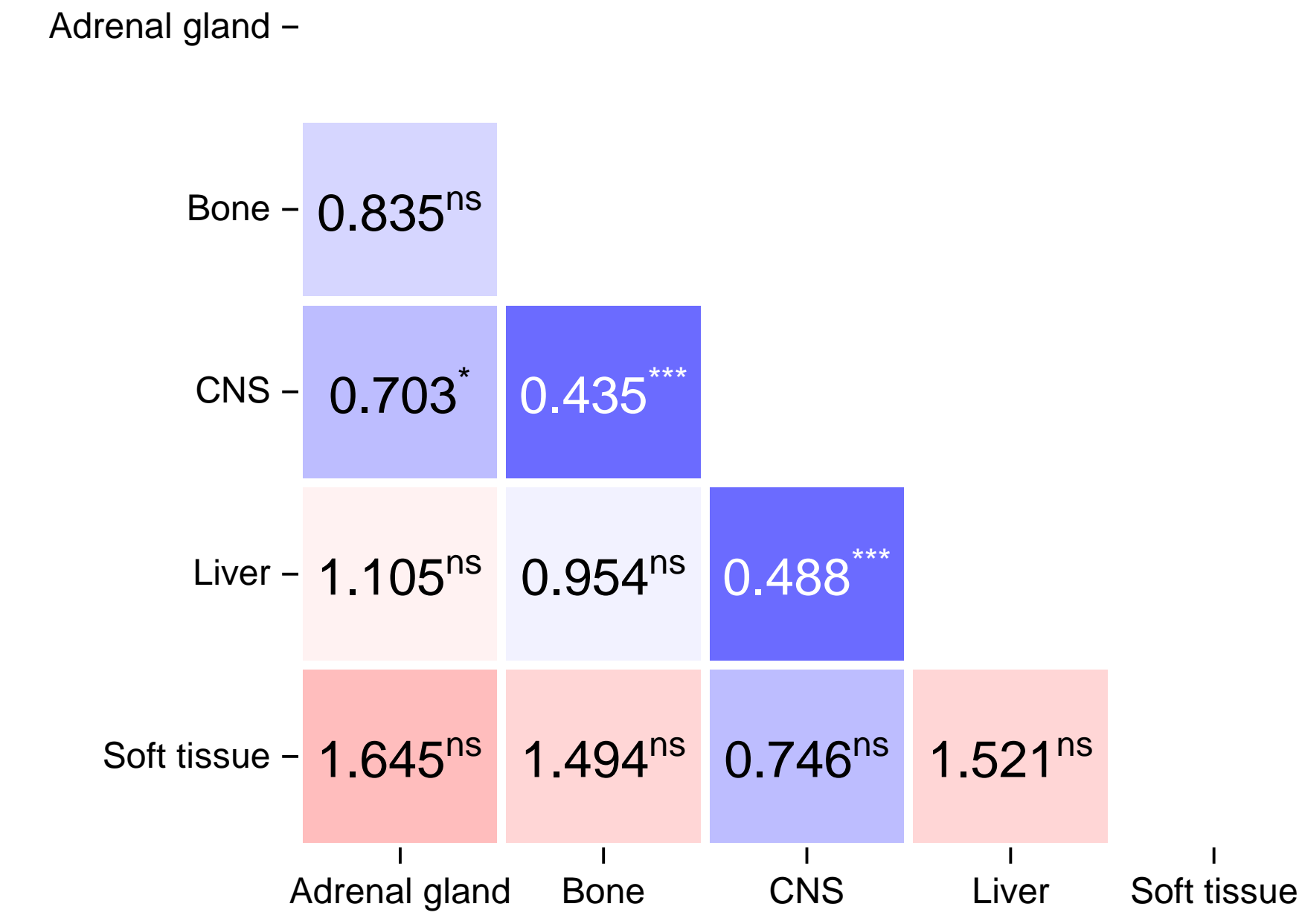

SCLC

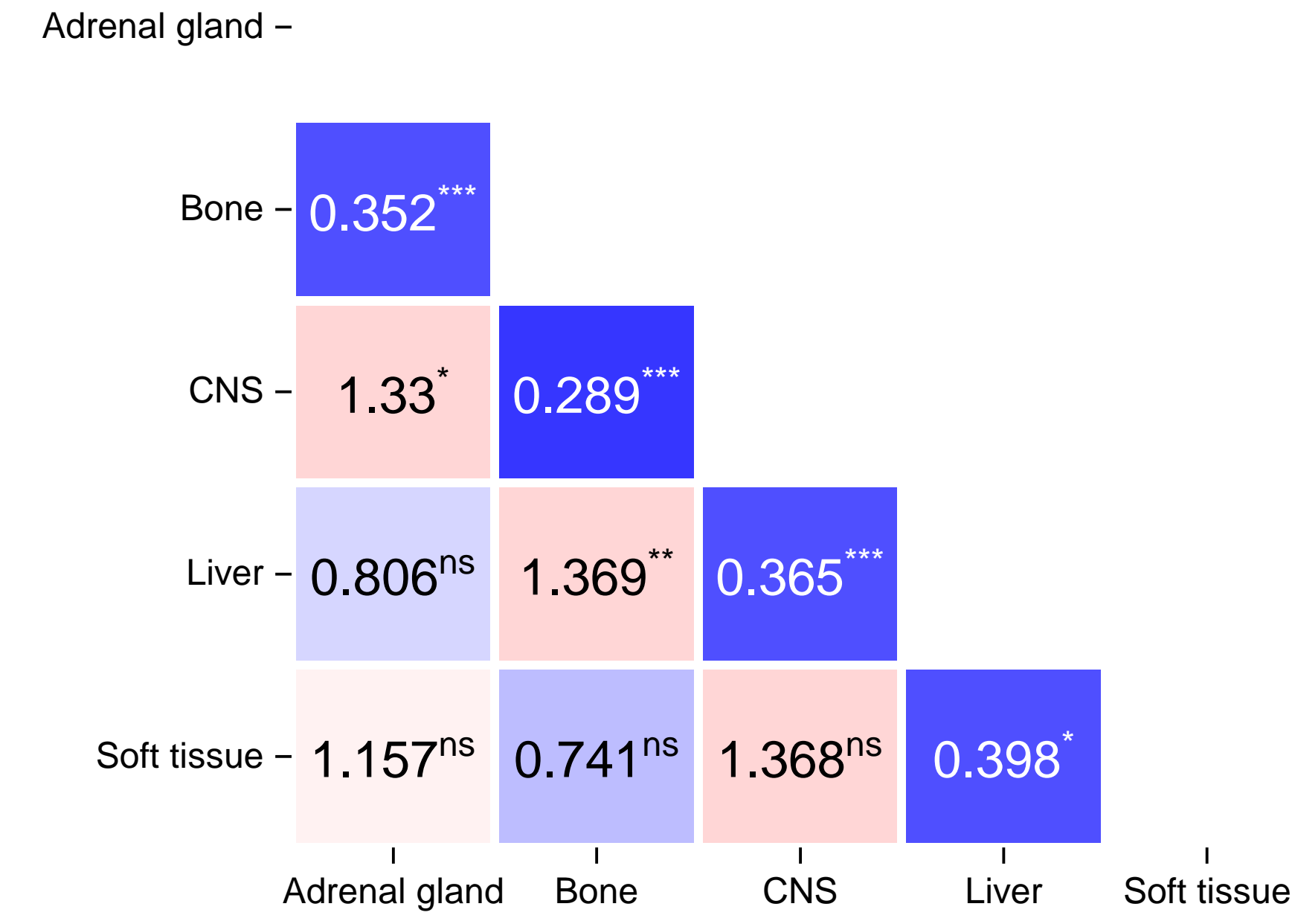

LCNEC

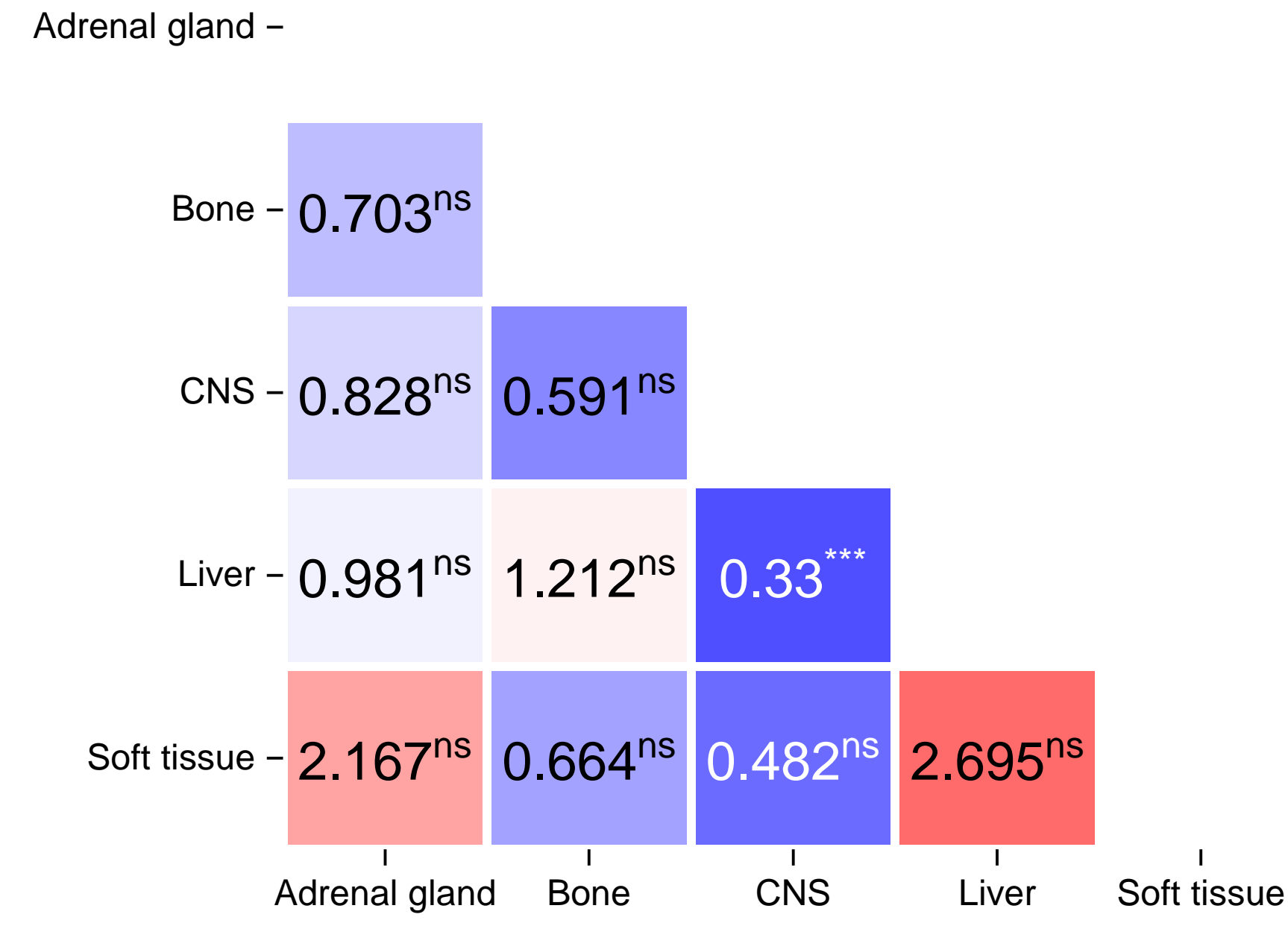

Total

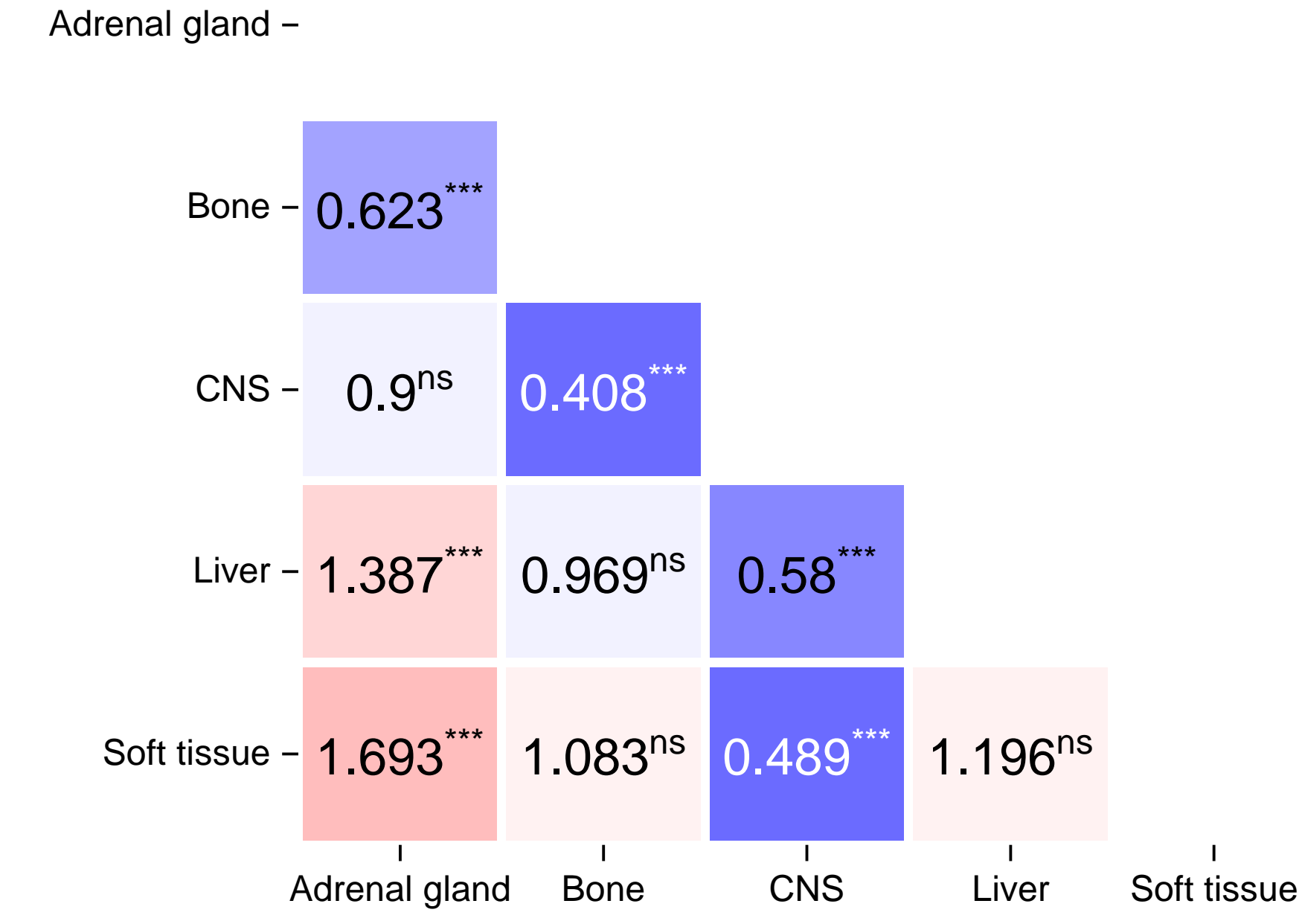

Odds ratio

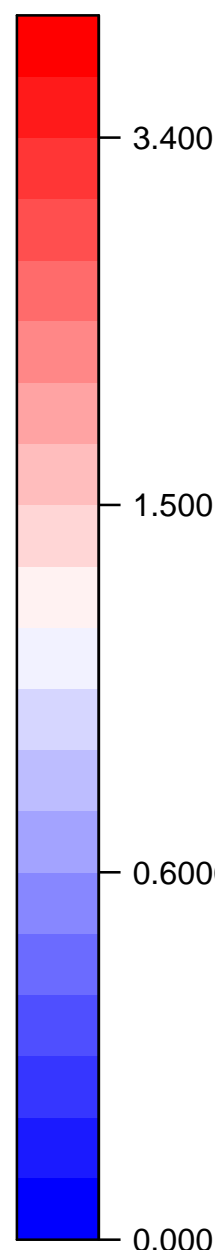

Supplement: Supplementary file 1 [file curroncol-29-00691-s001.zip › Figure S1.pdf]

Lung only

Lung and another carcinoma

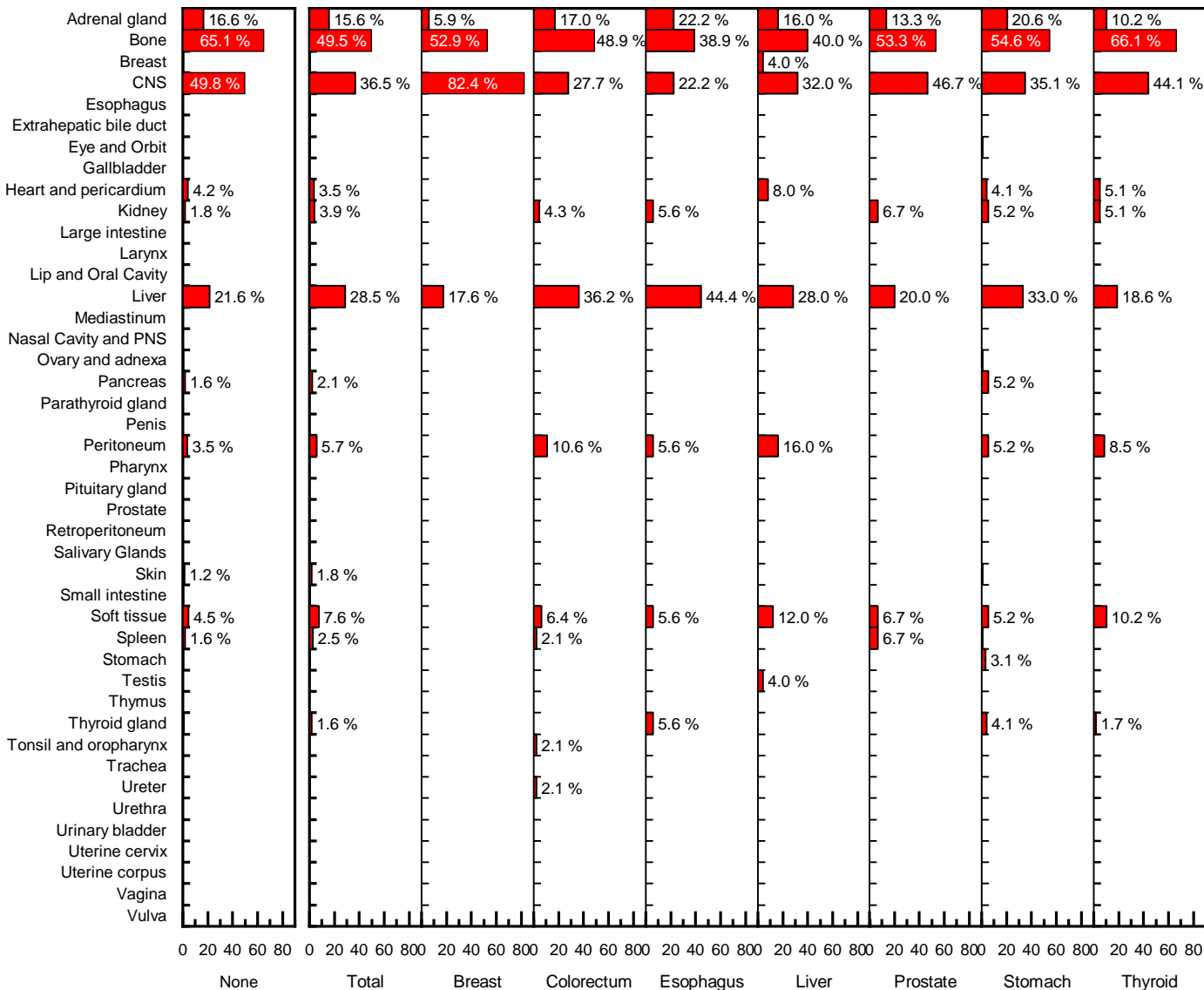

Supplement: Supplementary file 1 [file curroncol-29-00691-s001.zip › Figure S2.pdf]
